# A fast screening method for the detection of CERA in dried blood spots

**DOI:** 10.1002/dta.3142

**Published:** 2021-08-21

**Authors:** Angela Rocca, Laurent Martin, Tiia Kuuranne, Magnus Ericsson, Alexandre Marchand, Nicolas Leuenberger

**Affiliations:** ^1^ Swiss Laboratory for Doping Analyses, University Center of Legal Medicine, Lausanne and Geneva Lausanne University Hospital and University of Lausanne Lausanne Switzerland; ^2^ Analysis Department Agence Française de Lutte contre le Dopage (AFLD) Châtenay‐Malabry France

**Keywords:** anti‐doping, CERA, DBS, immunoassay

## Abstract

Continuous erythropoietin receptor activator (CERA) is a third‐generation erythropoiesis‐stimulating agent that was developed for the treatment of anemia. However, misuse of CERA for doping in endurance sports has been reported. Previous studies have shown blood as the matrix of choice for the detection of CERA, due to its high molecular weight. The use of dried blood spots (DBSs) for anti‐doping purposes constitutes a complementary approach to the standard urine and venous blood matrices and could facilitate sample collection and increase the number of blood samples available for analysis due to reduced costs of sample collection and transport. Here, we investigated whether CERA could be indirectly detected in extracts of single DBSs using an erythropoietin‐specific immunoassay that is capable of providing results within approximately 2 h. Reconstituted DBS samples were prepared from mixtures of red blood cell pellets and serum samples. The samples were collected in a previous clinical study in which six healthy volunteers were injected with a single, 200 μg dose of CERA. Using a commercially available ELISA kit, CERA was detected in the DBSs with a detection window of up to 20 days post‐injection. Furthermore, in order to demonstrate the fitness‐for‐purpose, three authentic doping control serum samples, which were identified as containing CERA, were analyzed by the presented methodological approach on DBS. The testing procedure described here could be used as a fast and cost‐effective method for the detection of CERA abuse in sport.

## 1 INTRODUCTION

Use of erythropoietin receptor agonists (ERAs)) for performance‐enhancing purposes can be detected via direct and indirect methods.^1^ Direct detection typically relies on separation techniques that depend on the isoelectric point (IEF method) or molecular weight (e.g., sodium *N*‐lauroylsarcosinate polyacrylamide gel electrophoresis [SAR‐PAGE] or SDS‐PAGE) of the ESA.^1^, ^2^

The pharmaceutical industry has developed recombinant human EPO (rhEPO) for therapeutic purposes.^3^ Epoetin alfa was the first rhEPO to become commercially available. The bioavailability of epoetin alfa is low, and up to three injections per week are needed to attain therapeutic effects.^4^ For doping, the short elimination time is advantageous, because the detection window is narrowed, whereas the effect is sustained. For this reason, the first generation rhEPO is the most used by cheated athletes. The optimal matrix to detect first generation of rhEPO is urine.^5^ Continuous erythropoietin receptor activator (CERA) also known as the drug MIRCERA® is a third‐generation ESA in which rhEPO is linked to a polyethylene glycol group. Compared with rhEPO, CERA has a lower affinity for the EPO receptor and a longer serum half‐life.^6^ In addition, the polyethylene glycol chain diminishes the renal filtration and elimination of CERA, making blood a more suitable matrix for its detection than urine.^7^, ^8^ The slow elimination time and long detection window may suggest that CERA is not the first choice of rhEPO for doping purposes. Nevertheless, recent anti‐doping operations have demonstrated that doping practices with CERA is still relevant scenario in endurance sport.^8^, ^9^ These findings indicate that doping is widespread in athletes competing regionally and that CERA is still a popular drug for endurance sports. They underline the need to facilitate blood sampling during nonmajor competitions.

Dried blood spots (DBSs) are convenient matrices for the analysis of blood samples. They are typically produced via a finger prick,^10^, ^11^ from where a drop of blood is collected onto a paper card. DBSs offer a number of advantages over conventional blood sample draw, including ease of collection by nonspecialized personnel, low invasiveness, reduced cost of analysis, facilitated transportation, easy storage, and high stability of analytes.^12^ Furthermore, previous studies have demonstrated a promising applicability and sufficient sensitivity of DBSs for the detection of a wide range of doping substances.^13^, ^14^, ^15^, ^16^ Recently, Reverter‐Branchat et al.^17^ described the methodology established for the monitoring of CERA in DBS by western‐blotting methods. These results support the concept of DBS‐based CERA testing in blood samples of elite athletes.

The aim of our current study was to determine if CERA can be detected in DBSs using a simple enzyme‐linked immunosorbent assay (ELISA). For this purpose, DBSs were generated from serum samples collected during a pilot study in which six healthy volunteers received a single intravenous injection of CERA (200 μg).

## 2 MATERIALS AND METHODS

### 2.1 DBS preparation

**FIGURE 1:**
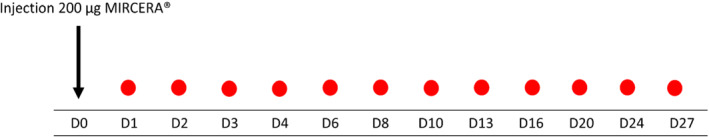
Experimental design of the CERA study. Volunteers were injected with a single 200 μg dose of CERA (MIRCERA®) on day 0 (D0). The red dots represent venous blood collection on post‐injection D1 through D27

This study used serum samples collected during a pilot excretion study performed by Lamon et al.,^18^ in which six healthy volunteers received a single intravenous injection of 200 μg CERA (MIRCERA®, Roche Pharma AG) and which was approved by the local ethical committee (protocol #05/08). The volunteers were Caucasian men aged 20–28 years, with a mean body mass index of 23.3 kg/m^2^ (SD 1.48 kg/m^2^). None of the volunteers was involved in elite sport. Blood samples were collected every morning over the first 4 post‐administration days. Thereafter, blood sampling was performed on days 6, 8, 10, 13, 16, 20, and 24. For four of the subjects, blood was also collected on day 27 post‐injection (Figure 1). Venous blood was collected from the elbow crease into EDTA tubes from heathy volunteers. After centrifugation at 300*g* for 10 min, the erythrocytes were isolated and washed twice with hypotonic solution (0.9% NaCl) and then mixed gently with an appropriate serum (50:50 ratio) as described by Reverter‐Branchat et al.^17^ Subsequently, 20‐μl aliquots of this modeled blood were spotted onto the DBS card (Whatman 903™ filter paper cards). The cards were left to dry for a minimum of 1 hour, and then the cards were stored at room temperature in sealed plastic bags until analysis. Same procedure was used for reconstituted volumetric absorptive microsampling (VAMS) (Neoteryx™).

### 2.2 DBS and VAMS extraction

For extraction, an entire blood spot of 20 μl was punched out of the DBS card manually and transferred to a 2‐ml Eppendorf tube. Alternatively the polymeric extremity of the VAMS with 20‐μl dried blood was directly put in a 2‐ml tube. Extraction buffer (1% Tween in 1‐ml H_2_O) was then added, and the sample was incubated for 15 min in a thermoshaker at 450 rpm and 37°C. Subsequently, the tube was centrifuged briefly and placed in a sonicator for 15 min. After sonication, the tube processed in the thermoshaker for another cycle of 15 min.

### 2.3 Immunoassays

ELISA assays from StemCell™ Technologies and R&D Systems were performed as described in manufacturer's instructions. Briefly, 100 μl of extracted DBS was added to the ELISA plate and detected by absorbance.

## 3 RESULTS

### 3.1 Quantification of EPO in DBSs using immunoassays

Immunoassays were used to determine the concentration of EPO in DBSs generated from serum samples collected from six volunteers after administration of a single dose (200 μg) of CERA. Blood samples were collected for up to 27 days (D0–D27, where D0 represents the day of administration; Figure 1). The cut‐off limit representing an abnormal concentration of EPO was 1.74 mIU/ml (14.62 pg/ml), as determined using the mean + 2SD value in serum samples from an unexposed to CERA population (*n* = 80) as described in Lamon et al.^18^

**FIGURE 2:**
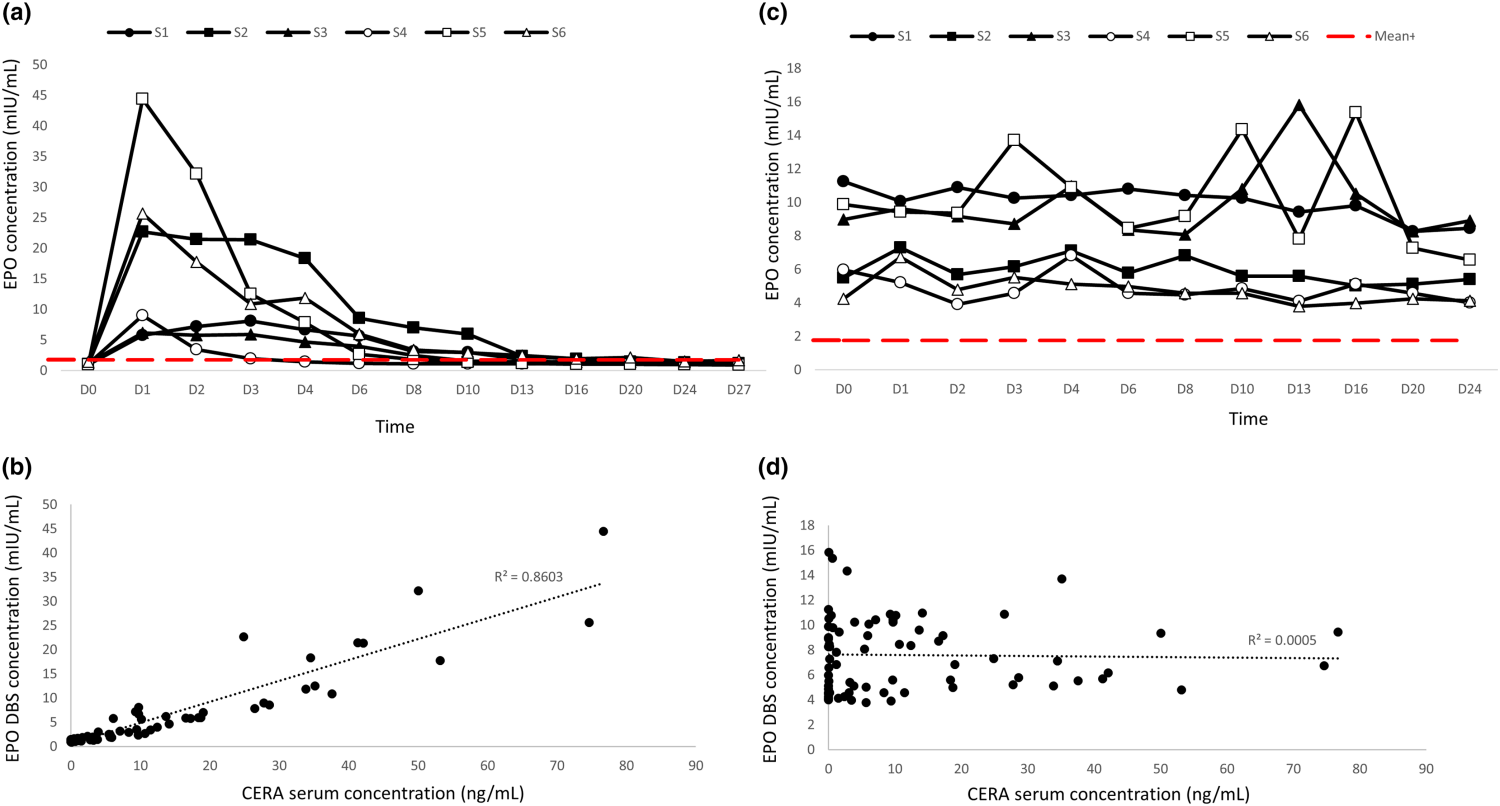
The concentrations of EPO in the DBS samples, as measured using two different EPO ELISA kits. (A) EPO concentrations measured using the EPO ELISA kit from StemCell™ technologies. (B) Pearson's correlation between the CERA concentration in serum (ng/ml)^14^ in DBSs (mIU/ml) by StemCell™ kit. (C) EPO concentrations by Quantikine IVD ELISA kit. (D) Pearson's correlation between the CERA concentration in serum (ng/ml)^14^ and the EPO concentration in DBSs (mIU/ml) measured using the human erythropoietin Quantikine IVD ELISA kit. In (a) and (C), the dashed red line represents the abnormal level cut‐off limit (1.74 mIU/ml, 14.62 pg/ml)

First, we used an ELISA kit from StemCell™ Technologies to detect EPO in the DBS samples. In this assay, the EPO concentration in the DBSs was observed to increase in all subjects following the CERA injection. Nevertheless, inter‐individual differences were significant as the detection time of EPO above the cut‐off limit ranged from three (subject 4) to 20 days (subject 6), the calculated mean being approximately 11 days (Figure 2A and Table 1). The overall dynamic trend of the changes in the levels of EPO in the DBSs was comparable to that of the level of CERA in the original serum samples from which the DBSs were generated.^18^ In addition, the concentrations of EPO in the DBSs and CERA in the original serum samples^18^ were highly correlated (Pearson's correlation coefficient: *R* = 0.9301, *p* < 0.001) (Figure 2B).

**TABLE 1:** Detection of abnormal concentrations of EPO in DBSs from each subject after injection of 200 μg CERA

ID	D0	D1–D3	D4	D6	D8	D10	D13	D16	D20	D24	D27
**S1**	**−**	+	+	+	+	+	+	**−**	**−**	**−**	**−**
**S2**	**−**	+	+	+	+	+	+	+	**−**	**−**	**−**
**S3**	**−**	+	+	+	+	**−**	**−**	**−**	**−**	**−**	
**S4**	**−**	+	**−**	**−**	**−**	**−**	**−**	**−**	**−**	**−**	
**S5**	**−**	+	+	+	+	**−**	**−**	**−**	**−**	**−**	**−**
**S6**	**−**	+	+	+	+	+	+	+	+	**−**	**−**

*Note*. Day 0 (D0) represents the day of injection. The + and – symbols represent concentrations of EPO that were higher or lower than the cut‐off limit (1.74 mIU/ml, 14.62 pg/ml), respectively. For subjects 3 and 4, the sampling stopped at D24.

For assay comparison, we measured the EPO concentrations in the DBSs using an ELISA kit from R&D Systems. Significant intra‐assay variation was observed regarding specificity, as the post‐injection concentrations of EPO detected using this kit were not consistent with the results obtained using the ELISA kit from StemCell™ Technologies. Moreover, a Pearson's correlation analysis did not show any association between the serum concentrations of CERA detected previously^18^ and the levels of EPO in the DBS samples detected using the R&D Systems kit (*R* = −0.02143, *p* > 0.05) (Figure 2D).

**FIGURE 3:**
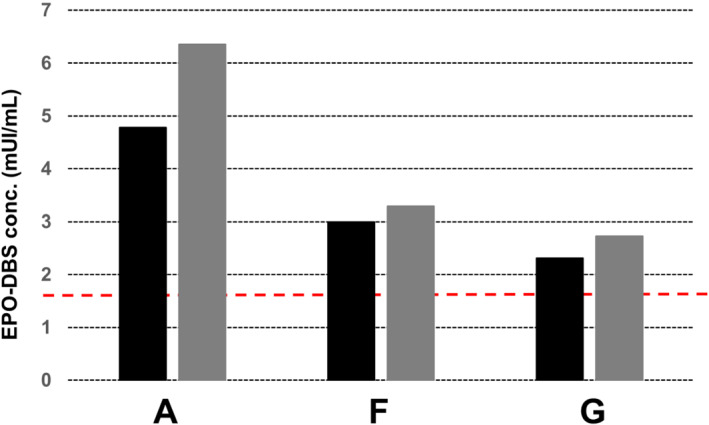
Analyses of EPO/CERA in DBSs generated from athletes serum samples. The serum samples tested (a, F, and G) were collected from elite subjects who tested positive for abnormal levels of CERA with SAR‐PAGE. EPO concentrations in DBSs (black) and VAMS (grey), as measured using the EPO ELISA kit from StemCell™ technologies. The dashed red line represents the abnormal level cut‐off limit (1.74 mIU/ml, 14.62 pg/ml)

In order to study the fitness‐for‐purpose of the presented DBS approach, three serum samples (A, F, and G) were reconstituted from authentic doping control serum samples confirmed previously by IEF and SDS‐PAGE to contain CERA.^8^ These samples were screened using the StemCell™ Technologies kit. Analysis using the StemCell™ Technologies ELISA kit revealed that the concentrations of EPO in samples A, F, and G were higher than the cut‐off limit established previously (1.72 mUI/ml;14.62 pg/ml) (Figure 3). Similar results were obtained with reconstituted VAMS (Figure 3).

## 4 DISCUSSION

Here, we describe the indirect detection of CERA in reconstituted DBSs using an EPO‐specific immunoassay (ELISA kit from StemCell™ Technologies), after a single injection of six volunteers with 200 μg CERA. Reassuringly, the dynamic trend of changes in the EPO concentration in the DBSs was similar to that of the CERA level in the original serum samples.^18^ In addition, there was a significant correlation (*R* = 0.9301, *p* < 0.05) between the EPO levels in the DBSs and the CERA levels in the serum samples. These findings suggest that DBSs could represent a potential alternative matrix for the initial testing of CERA for anti‐doping purposes.

In an earlier study, measurement of the endogenous EPO level in a negative control population has allowed for setting a cut‐off limit that was proposed as a threshold of EPO, below which samples would be considered negative (for doping). This limit, which was set as the mean value of the unexposed population plus 2SD, equated to 1.74 mIU/ml (14.62 pg/ml). The World Anti‐Doping Agency has recently set 25 pg/ml as the minimum required performance level for the detection of CERA in serum.^19^ Based on these preliminary results, the now described method complies with the criteria and shows approximately twice the sensitivity to detect EPO in DBS samples. Here, we used the determined cut‐off limit to establish the window of detection of EPO in each DBS sample. The window for CERA detection varied between the six subjects: the shortest detection time was until D3 after CERA injection (subject 4), whereas the longest prevailed until day D20 (subject 6). The earlier‐presented choice of mean + 2SD was made to achieve 95% confidence interval for assay specificity. Limited but existing risk of false suspicious can be considered acceptable, as the initial testing procedure is aimed at high sensitivity and whereas the non‐compromised specificity is assured at secondary stage by the confirmation method(s).

Our findings suggest that the detection of CERA in DBSs can be achieved using a specific EPO immunoassay. Primary screening of DBSs using this method could be used to provide preliminary information on the presence of CERA. Further investigations, such as western blot analyses, are needed for confirmation analysis of samples that exceed the EPO quantification cut‐off limit. The western‐blotting with DBS could be performed as already described by Reverter‐Branchat.^17^

Our exercise demonstrated the importance of assay selection, as the inter‐assay differences could be significant regarding the specificity for CERA. In addition to the ELISA kit from StemCell™ Technologies, we also attempted to measure the EPO concentration in DBSs using a specific immunoassay kit from R&D Systems. The results obtained with this kit were not consistent with those obtained using the kit from StemCell™ Technologies. In addition, they were not consistent with the CERA concentrations reported in the serum samples from which the DBSs were generated.^18^

DBS comparatively to starting from higher volumes of blood will logically be less sensitive with a potential risk of false negative for CERA.

WADA Guidelines recommend starting from 500 μl of serum to detect 25 pg/ml (MRPL) while these presented experiments were performed with 25‐fold less blood. However, even when starting from 20‐μl dried blood, our results indicate robust identification of 14.62 pg/ml. In this line, analysis of the reconstituted DBSs that were generated from three authentic doping control serum samples had been earlier observed on IEF/SDS‐PAGE to contain CERA. Moreover, the CERA in serum samples were identified with 1 ml of starting volume.

DBS testing could be used as a fast (2 h) screening method for the detection of CERA due to its ease of use over the collection of venous samples to compare standard western‐blotting method last several days from purification to results interpretation. Due the prolonged half‐life of CERA more frequent testing facilitated by the collect of DBS instead of venous blood could improve the efficiency and also deterrence from doping practices.

## 5 LIMITATIONS

This pilot study has clearly a number of limitations, which require further work prior to application to a routine context. Firstly, the number of volunteers is limited, and the samples used in the study were obtained from young Caucasian males who were not involved in elite sport. Secondly, an intravenous injection of 200‐μg MIRCERA® represents a therapeutic dose. The more probable doping scenario could be the application of micro‐doses that would be much more difficult to detect. Thirdly, DBS samples from clinical study with volunteers injected with high dose of Eprex were tested, and we did not observe significant increase with our method (data not shown). These data suggest that our strategy does not fit for first generation recombinant EPOs. Finally, the reconstitution of DBSs results in dilution of the total amount of CERA in the sample, leading to a probable deviation from the real serum concentration.

## 6 CONCLUSION

In conclusion, a single injection of 200‐μg CERA was detectable in DBS samples using a specific EPO immunoassay. Notably, the specific ELISA kit used for the analysis influenced the results. The ELISA kit from StemCell™ Technologies showed good performance, whereas the specificity of the immunoassay kit from R&D Systems was not fit for this purpose. Based on our findings, we suggest that an approach based on immunoassay analysis of DBS samples has a potential to be developed as a simple and rapid method for a primary screening of CERA misuse. For this specific use, DBS could be a valuable addition to urine samples, that will stay the matrix of choice to test the other rhEPOs but is not ideal for CERA detection. A western blot analysis would be required for confirmation procedure if the EPO concentration detected in the immunoassay exceeded a predefined cut‐off limit as described as Reverter‐Branchat et al.^17^
